# Records of *Wenchengia* (Lamiaceae) from Vietnam

**DOI:** 10.3897/BDJ.4.e9596

**Published:** 2016-08-03

**Authors:** Alan Paton, Peter B Phillipson, Somran Suddee

**Affiliations:** ‡ The Royal Botanic Gardens, Kew, London, United Kingdom; § Missouri Botanical Garden, St. Louis, United States of America; | Institut de Systématique, Évolution et Biodiversité (UMR 7205 - CNRS MNHN UPMC EPHE), Muséum national d'Histoire naturelle, Sorbonne Universités, Paris, France; ¶ The Forest Herbarium, Department of National Parks, Wildlife and Plant Conservation, Bangkok, Thailand

**Keywords:** Hainan, endemic, Bach Ma National Park, Ba Na Hills

## Abstract

**Background:**

The monotypic genus *Wenchengia* (Lamiaceae) has been thought to be endemic to Hainan, China. This paper reports on historic records of *Wenchengia alternifolia* collected from Vietnam. The recent recuration and modernisation of the Paris herbarium greatly facilitated this discovery.

**New information:**

During preparatory work supporting the account for the Lamiaceae of the Flora of Thailand, three specimens of *Wenchengia* from central Vietnam were found in the Herbarium of the Musuem National d'Histoire Naturelle in Paris (P), and subsequently two duplicates were found in the Herbarium at Kew (K, abbreviations following Thiers 2016). The specimens were collected in and before 1927 and it is not known if the species is still extant in Vietnam. Searches for extant populations should focus in the Ba Na Hills or Bach Ma National Park, central Vietnam.

## Introduction

The monotypic genus *Wenchengia* C.Y. Wu & S. Chow has hitherto been recorded as endemic to Hainan (Li et al. 2014, Francisco-Ortega et al. 2010, Manchester et al. 2009). *Wenchengia alternifolia* C.Y. Wu & S. Chow was originally described on the basis of two collections from Hainan dating from the 1930s (Wu and Chow 1965). Very few other specimens of the species exist (Li et al. 2012)

Li et al. (2012) record a rediscovery of the species from Shuangximu Valley, Wanning, Hainan Province of China, where it was recorded at two adjacent sites growing the shade of forest along moist stone cracks and cliffs near a waterfall at around 100–300 m altitude. They provide a full and richly illustrated account of these populations' morphology. They also place the genus in a basal position within subfamily Scutellarioideae of the Lamiaceae based on rbcL sequence data. In a further study Li et al. (2014) characterise the species as Critically Endangered using IUCN Redlist Catergories and Criteria v 3.1 (IUCN 2001).

The aim of this paper is to draw attention to three collections of *Wenchengia alternifolia* from Vietnam. These specimens are housed in the Herbarium of the Muséum National d'Histoire Naturelle whose recent renovation and recuration of specimens into systematic order greatly facilitated the discovery of these specimens. Duplicates of two of these specimens have also now been found at Kew.

## Materials and methods

The specimens of *Wenchengia* from Vietnam were discovered during preparatory work for the Flora of Thailand Lamiaceae account, and examination of specimens of *Scutellaria* L. and indetermined Lamiaceae housed in P. The description below is based on the Vietnam material and the detailed observations of Li et al. (2014), Li et al. (2012), Cantino and Abu-Asab (1993), material in MO from Hainan, and on an isotype of *Wenchengia alternifolia* also identified in the herbarium in Paris, which was previously determined as *Scutellaria* sp. The text in parentheses in the description below identifies the slight morphological differences between the Vietnam material and that from Hainan.

## Taxon treatments

### Wenchengia alternifolia

C.Y.Wu & S.Chow 1965

urn:lsid:ipni.org:names:461897-1:1.2.2.1.1.1

#### Materials

- **Type status:** Isotype. **Occurrence:** catalogNumber: P04158076; recordNumber: 73689; recordedBy: F.C. How; **Taxon:** scientificName: Wenchengia alternifolia; **Location:** country: China; stateProvince: Hainan; locality: Boating; verbatimLocality: Po-ting; **Identification:** identifiedBy: Alan Paton; dateIdentified: 2016; **Event:** year: 1938; month: 9; day: 23; **Record Level:** institutionCode: P; basisOfRecord: PreservedSpecimen; source: http://coldb.mnhn.fr/catalognumber/mnhn/p/p04158076
- **Type status:** Other material. **Occurrence:** catalogNumber: P03006424; recordNumber: 3376; recordedBy: J. & M.S. Clemens; **Taxon:** scientificName: Wenchengia alternifolia; **Location:** country: Vietnam; stateProvince: Da Nang; locality: Ba Na Hills; verbatimLocality: Mt Bani, about 25 km from Tourane; **Identification:** identifiedBy: Somran Suddee; dateIdentified: 2001; **Event:** year: 1927; month: 6; verbatimEventDate: 4-13 June 1927; **Record Level:** institutionCode: P; basisOfRecord: PreservedSpecimen; source: http://coldb.mnhn.fr/catalognumber/mnhn/p/p03006424
- **Type status:** Other material. **Occurrence:** catalogNumber: P04442939; recordNumber: 3882; recordedBy: J. & M.S. Clemens; **Taxon:** scientificName: Wenchengia alternifolia; **Location:** country: Vietnam; stateProvince: Da Nang; locality: Ba Na Hills; verbatimLocality: Mt Bani, about 25 km from Tourane; **Identification:** identifiedBy: Alan Paton; dateIdentified: 2016; **Event:** year: 1927; month: 7; verbatimEventDate: Jul-1927; **Record Level:** institutionCode: P; basisOfRecord: PreservedSpecimen; source: http://coldb.mnhn.fr/catalognumber/mnhn/p/p04442939
- **Type status:** Other material. **Occurrence:** catalogNumber: P03006423; recordNumber: 1516; recordedBy: P.A. Eberhardt; **Taxon:** scientificName: Wenchengia alternifolia; **Location:** country: Vietnam; stateProvince: Thua Thien-Hué; locality: Hai-Mit; verbatimLocality: Thua Thien Province Hai-Mit; **Identification:** identifiedBy: Somran Suddee; dateIdentified: 2001; **Event:** verbatimEventDate: no date; **Record Level:** institutionCode: P; basisOfRecord: PreservedSpecimen; source: http://coldb.mnhn.fr/catalognumber/mnhn/p/p03006423
- **Type status:** Other material. **Occurrence:** catalogNumber: P03006423; recordNumber: 1516; recordedBy: P.A. Eberhardt; **Taxon:** scientificName: Wenchengia alternifolia; **Location:** country: Vietnam; stateProvince: Thua Thien-Hué; locality: Hai-Mit; verbatimLocality: Thua Thien Province Hai-Mit; **Identification:** identifiedBy: Somran Suddee; dateIdentified: 2001; **Event:** verbatimEventDate: no date; **Record Level:** institutionCode: P; basisOfRecord: PreservedSpecimen; source: http://coldb.mnhn.fr/catalognumber/mnhn/p/p03006422
- **Type status:** Other material. **Occurrence:** catalogNumber: K001053643; recordNumber: 3376; recordedBy: J. & M.S. Clemens; **Taxon:** scientificName: Wenchengia alternifolia; **Location:** country: Vietnam; stateProvince: Da Nang; locality: Ba Na Hills; verbatimLocality: Mt Bani, about 25 km from Tourane; **Identification:** identifiedBy: Alan Paton; dateIdentified: 2016; **Event:** year: 1927; verbatimEventDate: May to July 1927; **Record Level:** institutionCode: K; basisOfRecord: PreservedSpecimen; source: http://specimens.kew.org/herbarium/K001053643
- **Type status:** Other material. **Occurrence:** catalogNumber: K001053644; recordNumber: 3882; recordedBy: J. & M.S. Clemens; **Taxon:** scientificName: Wenchengia alternifolia; **Location:** country: Vietnam; stateProvince: Da Nang; locality: Ba Na Hills; verbatimLocality: Mt Bani, about 25 km from Tourane; **Identification:** identifiedBy: Alan Paton; dateIdentified: 2016; **Event:** year: 1927; verbatimEventDate: May to July 1927; **Record Level:** institutionCode: K; basisOfRecord: PreservedSpecimen; source: http://specimens.kew.org/herbarium/K001053644
- **Type status:** Other material. **Occurrence:** catalogNumber: 50063593; recordNumber: 9017; recordedBy: Hu Chi Ming; **Taxon:** scientificName: Wenchengia alternifolia; **Location:** country: China; stateProvince: Hainan; verbatimLocality: Hainan; **Identification:** identifiedBy: Alan Paton; dateIdentified: 2016; **Event:** year: 1996; month: 7; day: 10; **Record Level:** institutionCode: MO; basisOfRecord: PreservedSpecimen; source: http://www.tropicos.org/Image/100124013

#### Description

**Figure 1.**
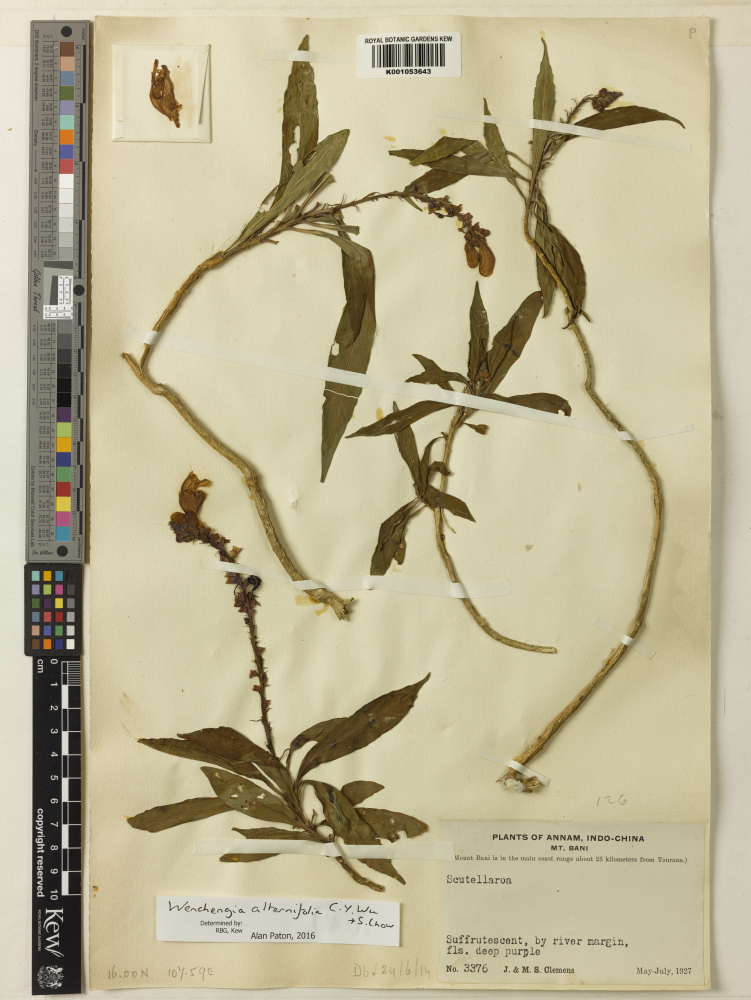
Wenchengia alternifolia *J. & M.S. Clemens* 3376 (K).

Subshrubs 30–60 cm tall (15–40 cm in Hainan) with stems ascending, prostrate at base, leafless in lower parts with elevated reniform scars, hirtellous with short antrorse or patent eglandular hairs. Leaves alternate, rarely subopposite; blades usually oblanceolate to narrow elliptic (only oblanceolate in Hainan), 50–90 mm long, 5–28 mm broad (15–40 mm broad in Hainan) margins shallowly undulate to almost entire (only undulate in Hainan), base cuneate or narrowly cuneate and decurrent, apex obtuse to acuminate, abaxially hirtellous with short eglandular hairs on veins and margins, adaxially glabrous, glandular punctate on both surfaces; petioles 5–13 mm long. Inflorescences single, unbranched and terminal or with two or three unbranched inflorescences arising from the upper leaves (unbranched in Hainan) with single flowers spirally arranged but facing in one direction and subtended by a linear bract 3–5 mm long; bracteoles soon deciduous; pedicels 2 mm; inflorescence axis and pedicels hirtellous with short eglandular hairs and scattered sessile glands. Calyx purplish, 6 mm long, and 2-lipped with five shallow teeth, hirtellous with short eglandular hairs and scattered sessile glands; the upper lip with three equal, deltoid teeth; the lower lip is formed by two dilated and coalescent teeth, nearly truncate, just less than the length of the upper lip (difference in lip length is greater in Hainan material, but the Vietnamese material seen is less mature). Corolla , blue to deep purple (white also recorded in Hainan), tubular-campanulate, 2-lipped, 2.0–3.0 cm long, and sparsely covered by capitate glandular and nonglandular hairs; tube arcuate, with scattered hairs (rather than bearded in Hainan) at the middle inside, and 1.6–2.4 cm long with a narrow, bent base and a gradually dilated broad throat ranging in width from 6.0–8.5 mm; upper lip 2-lobed, 4.5–5.0 mm wide, and slightly concave; lower lip is 3-lobed,5.0–6.0 mm wide, subelliptic, and spreading. Ovary lobed for a quarter of its length, the style arising three-quarters of the way from the ovary base to its apex, pubescent and glandular in upper half; style branches unequal (Cantino and Abu-Asab 1993) Fruit not seen; see Li et al. (2012) for fruit description. Pollen tricolpate, spherical to subprolate; exine is tectate-perforate and suprareticulate; in thin section, the endexine is thin, the foot layer discontinuous, the columellae are simple, and the tectum is discontinous (Cantino and Abu-Asab 1993). Fig. 1

##### Habitat in Vietnam

Banks of forest trail or river. Altitude not recorded.

## Discussion

It is perhaps not surprising that *Wenchengia* is not endemic to Hainan. Francisco-Ortega et al. (2010), reporting seven endemic genera, anticipated that many species endemic to Hainan would lose their endemic status once the floras of Vietnam and South China were better studied, and Zhu et al. (2003) report a similarity coefficient at generic level of 90.8% between the flora of Vietnam and Hainan. What is more surprising is that the three collections all predate the collection of the type and paratype from Hainan, but no record of *Wenchengia* in Vietnam has been documented (Phuong 2000). The Clemens specimens were collected in 1927. The P.A. Eberhardt collection has no collection date, but he is known to have collected in Vietnam between 1906 and 1920 (Lecomte 1944).

This discovery of *Wenchengia* in Vietnam suggests that the Hainan populations are a relict of a once, and perhaps still, wider distribution. *Wenchengia* lies at the base of a clade including *Holmlskioldia* Retz. which is in turn is sister to *Tinnea* Kotschy & Peyr., *Renschia* Vatke and *Scutellaria*. Whereas *Scutellaria* is subcosmopolitan, *Tinnea* is African, *Renschia* endemic to N. Somalia, and *Holmskolidia* is found from the Himalaya to Myanmar (Cantino 1992, Harley et al. 2004).

The Vietnamese populations are morphologically similar to those from Hainan, the main differences being vegetative. The character variation is similar to the sorts of infraspecific variation seen in species of *Scutellaria*. The populations in Hainan are small, and given the few collections in Vietnam, any populations there are likely also to be small. A level of genetic drift between the populations might be expected, but genetic study of the populations is required to further investigate this. It is not known whether the Vietnamese populations are still extant, and the exact localities of the collections are unclear. There has been a lot of recent development in the tourist industry in the Ba Na Hills, Da Nang Province, which has had a tourist resort since 1919 and now hosts the worlds longest cable car system (Anon. 2016). The Eberhardt locality, 'Hoi-Mit' in Thua Than-Hue Province has not been traced. It is possible that the locality is now in, or close to, the Bach Ma National Park, close to Ba Na, from where recently a new species of *Callicarpa*, *Callicarpa bachmaensis* Soejima & Tagane, was discovered growing in a similar habitat to *Wenchengia* (Soejima et al. 2016). Efforts to search for extant populations could focus in Bach Ma in addition to the Ba Na Hills, in rocky river or path banks within forest.

## Supplementary Material

XML Treatment for Wenchengia alternifolia
